# Nomograms to estimate long‐term overall survival and tongue cancer‐specific survival of patients with tongue squamous cell carcinoma

**DOI:** 10.1002/cam4.1021

**Published:** 2017-04-14

**Authors:** Yun Li, Zhenyan Zhao, Xiaoxiao Liu, Jun Ju, Juan Chai, Qianwei Ni, Chao Ma, Tao Gao, Moyi Sun

**Affiliations:** ^1^ State Key Laboratory of Military StomatologyNational Clinical Research Center for Oral DiseasesShaanxi Clinical Research Center for Oral DiseasesDepartment of Oral and Maxillofacial SurgerySchool of StomatologyFourth Military Medical UniversityXi'anChina; ^2^ Department of StomatologyFengtai HospitalPeking University First HospitalBeijingChina; ^3^ Department of Otolaryngology Head Neck SurgeryNavy General HospitalBeijingChina

**Keywords:** Head and neck, nomogram, overall survival, tongue cancer‐specific survival, tongue squamous cell carcinoma

## Abstract

The aim of this study was to construct nomograms to predict long‐term overall survival (OS) and tongue cancer‐specific survival (TCSS) of tongue squamous cell carcinoma (TSCC) patients based on clinical and tumor characteristics. Clinical, tumor, and treatment characteristics of 12,674 patients diagnosed with TSCC between 2004 and 2013 were collected from the Surveillance, Epidemiology, and End Results database. These patients were then divided into surgery and nonsurgery cohorts, and nomograms were developed for each of these groups. The step‐down method and cumulative incidence function were used for model selection to determine the significant prognostic factors associated with OS and TCSS. These prognostic variables were incorporated into nomograms. An external cohort was used to validate the surgery nomograms. Seven variables were used to create the surgery nomograms for OS and TCSS, which had c‐indexes of 0.709 and 0.728, respectively; for the external validation cohort, the c‐indexes were 0.691 and 0.711, respectively. Nine variables were used to create the nonsurgery nomograms for OS and TCSS, which had c‐indexes of 0.750 and 0.754, respectively. The calibration curves of the 5‐ and 8‐year surgery and nonsurgery nomograms showed excellent agreement between the probabilities and observed values. By incorporating clinicopathological and host characteristics in patients, we are the first to establish nomograms that accurately predict prognosis for individual patients with TSCC. These nomograms ought to provide more personalized and reliable prognostic information, and improve clinical decision‐making for TSCC patients.

## Introduction

Squamous cell carcinoma (SCC) is the most common type of head and neck cancer, with tongue squamous cell carcinoma (TSCC) accounting for more than half of oral cavity cancers 1, 2, 3, 4. Rapid local invasion and early lymph node metastasis are the most notorious features of TSCC, which usually causes malfunction of mastication, speech, and deglutition, resulting in poor survival and quality of life 5, 6, 7, 8. Although the diagnostic techniques and multimodal therapy management methods have greatly advanced in recent years, the 5‐year overall survival (OS) of TSCC patients is less than 50% owing to their diverse clinicopathological characteristics 2, 9, 10, 11.

Currently, the American Joint Committee on Cancer (AJCC) TNM staging classification is used to determine treatment strategies for TSCC patients 12. This classification system is an objective and accurate tool that is used to predict prognosis for an entire population of patients. However, it is ineffective for predicting outcomes in an individual patient, because of its inability to take into account the role of other tumor factors and important host characteristics such as age, gender, tobacco use, as well as tumor variables such as tumor size and tumor pathological type 13, 14. A tool that incorporates these factors to accurately predict patients’ outcomes is required.

Nomograms are a reliable and convenient statistical prediction tool with the ability to estimate events in an individual patient via combining numerous elements that are distinct from the widely accepted TNM factors 15. To the best of our knowledge, nomograms have been widely used for a host of solid tumors including breast cancer 16, gastric carcinoma 17, and head and neck carcinomas 18, 19, 20, 21. To date, nomograms for predicting prognoses of individual patients with TSCC, which is considered a biologically distinct entity from cancers afflicting other sites of the head and neck, have not been reported.

In this study, which is based on multi‐institution and multi‐population data from the Surveillance, Epidemiology, and End Results (SEER) database, we aimed to establish the first comprehensive and practical TSCC nomograms that predict OS and tongue cancer‐specific survival (TCSS) for patients undergoing surgical treatment as well as those not surgically treated. Such competing risk nomograms will assist clinicians by providing more personalized and precise prognostic information in clinical practice.

## Materials and Methods

### Patients

Our study data were collected from two institutions: the SEER program of the National Cancer Institute, which currently collects and publishes cancer incidence and survival data from population‐based cancer registries 22 and a Chinese public medical institution (the Department of Oral and Maxillofacial Surgery, School of Stomatology, Fourth Military Medical University).

**Figure 1:**
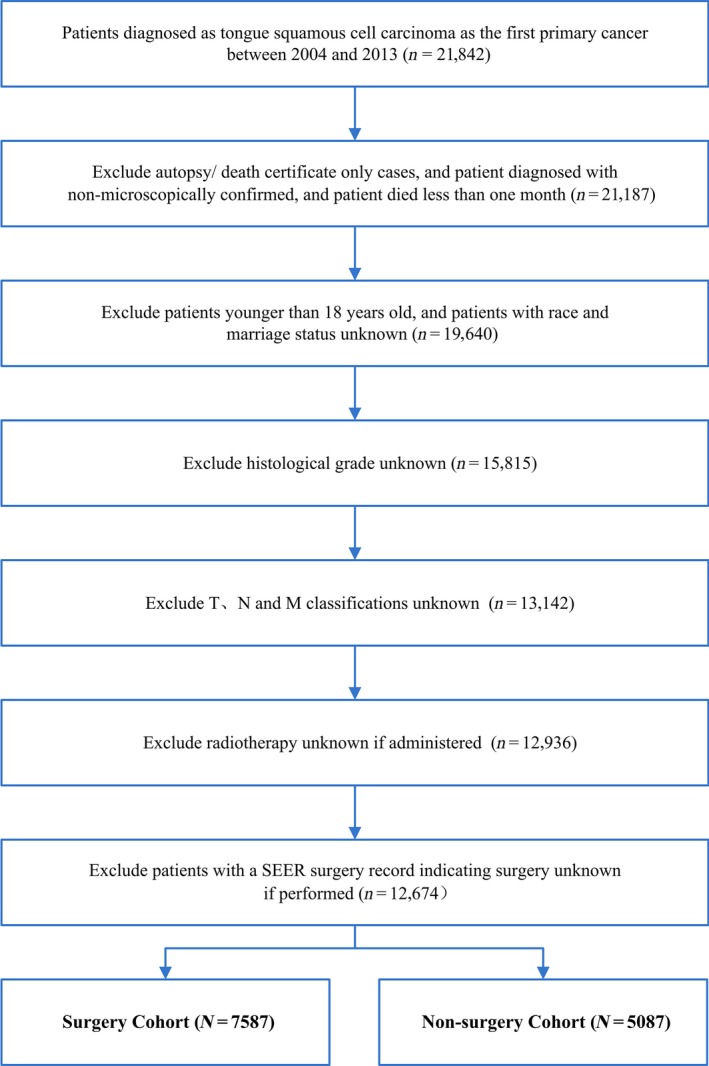
Flowchart for the Surveillance, Epidemiology, and End Results data selection.

Patients diagnosed with TSCC as the only primary cancer between 2004 and 2013 were extracted from the SEER program of the National Cancer Institute and included in the study cohort for retrospective analysis and review. The data selection process is shown in Figure 1. The inclusion criteria were as follows: the diagnosis was histologically confirmed, the tumor had malignant behavior, the patients were older than age 18, clinical and pathologic characteristics (age at diagnosis, race, sex, marital status, grade, TNM status, surgery, and radiotherapy) were known and detailed, and active follow‐up was maintained. Patients whose diagnostic information was obtained only from a death certificate or autopsy report, as well as those who died within 1 month of surgery, were excluded. The eligible patients were further classified into surgery and nonsurgery cohorts to develop distinct individual nomograms.

With respect to race, American Indian/Alaskan Native and Asian/Pacific Islanders were recorded as “other”. Tumor grade classification was as follows: I (well differentiated), II (moderately differentiated), III (poorly differentiated), IV (undifferentiated). Marital status was classified as U (unmarried) and M (married). Pathologic TNM status was determined using the AJCC Staging Manual (7th edition) 23. Continuous variables such as age at diagnosis were transformed into categorical variables based on the SEER program. All covariates were analyzed as categorical variables.

Using the same inclusion and exclusion criteria, another independent cohort of consecutive patients treated at a Chinese public medical institution (the Department of Oral and Maxillofacial Surgery, School of Stomatology, Fourth Military Medical University) between 2007 and 2013 served as an external validation cohort.

The study was approved by our Institutional Ethics Committees. Informed consent was not required for data obtained from the SEER database.

### Statistical analyses and construction of the nomogram

The surgery and nonsurgery cohorts collected from the SEER database were used to conduct prognostic analysis and develop nomograms. Nine clinical and pathologic characteristics, including age at diagnosis, race, sex, marital status, grade, TNM status, and radiotherapy, were used to conduct the analyses.

The median follow‐up time was estimated as the actual patient survival time. OS was defined as the time from diagnosis to death or censoring. The Kaplan–Meier method and log‐rank test were applied for calculating and assessing the difference in prognostic factors of OS. Possible prognostic factors (*P* < 0.001) associated with OS were subjected to a multivariable Cox proportional hazards analysis. The independent prognostic factors on multivariate analysis were used to build nomograms for OS.

When constructing a competing risks nomogram for TCSS, death from tongue squamous cancer and death from other causes were considered as two separate events. TCSS was defined as the time from diagnosis to death attributed to TSCC, date of last follow‐up, or December 31, 2013 (if the date of last follow‐up was after 2013). The cumulative incidence function was used to assess the probability of death, and the difference was evaluated by Gray's test 24. A proportional subdistribution hazards regression method proposed by Fine and Gray was used to set up the competing risks model 25. Integrating all the associated risk factors (*P* < 0.001), nomograms were developed to predict the risk of death and cancer‐specific mortality in 5 and 8 years.

### Validation and calibration of the nomogram

Both surgery and nonsurgery nomograms were subjected to internal validation; the surgery nomograms were also subjected to validation using an external validation cohort. Two methods, discrimination and calibration, were used to conduct both internal and external validation for estimating the predictive accuracy of the model. Discrimination was quantified by the probability of concordance (c‐index), values ranged from 0.5 (no discrimination) to 1.0 (perfect discrimination) as proposed by Harrell 26. All internal validations were performed using bootstrapping with 1,000 resamples. A marginal estimate versus actual model was used to plot calibration curves that represented the predictive accuracy of the nomograms. A 45‐degree diagonal line was the ideal target.

All statistics analyses were conducted using the SPSS software (19.0, Chicago, IL) and R software version 3.3.0 (http://www.r-project.org/) with R packages, rms and cmprsk. All calculated *P* values were two‐sided, and *P* < 0.001 was considered significant.

## Results

### Baseline characteristics

A total of 21,842 patients diagnosed with TSCC as the sole primary cancer between 2004 and 2013 were extracted from the SEER database; 9,168 patients were excluded because of unknown clinical and pathologic information or because the patients died within 1 month. Finally, 7,587 and 5,087 patients comprised the surgery and nonsurgery cohort, respectively. Demographic information of the patients as well as their tumor clinicopathological characteristics are listed in Table 1. The median follow‐up periods for surgery cohort and nonsurgery cohort were 31 (range, 1–119) and 25 (range, 1–119) months, and the median ages were 59.0 (range, 18–102), and 61.0 (range, 20–102) years, respectively. The majority of the patients in both cohorts were male (62.0% and 82.8%, respectively), Whites (85.7% and 87.0%, respectively), and married (70.4% and 66.0%, respectively).

**Table 1:** Clinical and tumor characteristics

Variables	Surgery cohort (*n* = 7587)		Nonsurgery cohort (*n* = 5087)		Validation cohort (*n* = 191)	
	No.	%	No.	%	No.	%
Age^a^ (years)						
18–44	985	13.0	209	4.1	51	26.7
45–54	1714	22.6	1126	22.1	43	22.5
55–64	2287	30.1	1952	38.4	44	23.0
65–74	1522	20.1	1175	23.1	35	18.3
75+	1079	14.2	625	12.3	18	9.5
Race						
White	6499	85.7	4428	87.0	0	0
Black	416	5.4	469	9.2	0	0
Other^b^	672	8.9	190	3.7	191	100
Sex						
Female	2882	38.0	875	17.2	91	47.6
Male	4705	62.0	4212	82.8	100	52.4
Marital status						
Unmarried	2245	29.6	1732	34.0	23	12.0
Married	5342	70.4	3355	66.0	168	88.0
Grade						
I	1621	21.4	345	6.8	92	48.2
II	4047	53.3	2289	45.0	66	34.6
III	1877	24.7	2390	47.0	29	15.2
IV	42	0.6	63	1.2	4	2.1
T stage						
T1	3905	51.5	907	17.8	104	54.5
T2	2389	31.5	2142	42.1	59	30.9
T3	706	9.2	860	16.9	11	5.8
T4a	557	7.3	1035	20.4	12	6.3
T4b	30	0.5	143	2.8	5	2.5
N stage						
N0	4528	59.6	957	18.8	158	82.7
N1	1228	16.2	1090	21.4	18	9.4
N2a	257	3.4	380	7.5	4	2.1
N2b	1152	15.2	1266	24.9	6	3.2
N2c	331	4.4	1128	22.2	3	1.6
N3	91	1.2	266	5.2	2	1.0
M stage						
M0	7527	99.2	4888	96.1	186	97.4
M1	60	0.8	199	3.9	5	2.6
Radiation						
Yes	3606	47.5	4491	88.3	107	56.0
None	3981	52.5	596	11.7	84	44.0
Surgery performed						
Yes	7587	100	0	0	191	100
None	0	0	5087	100	0	0

a Age at diagnosis.

b Other including American Indian/AK Native, Asian/Pacific Islander.

Of 7,587 patients in the surgery cohort, the majority of tumors were moderately differentiated (4,047, 53.3%), T1–T2 status (6,294, 83.0%) had no positive neck nodes (4,528, 59.6%) and had no distant metastases (7,527, 99.2%). All patients underwent surgery and 47.5% received radiotherapy. There were 2,409 patients (31.8%) who had died by the end of the follow‐up period; 1,703 (22.5%) died of TSCC, while 706 (9.3%) died of other causes.

In the nonsurgery cohort, moderately and poorly differentiated tumors accounted for 92.0% (4,679). T1–T2 status, positive neck nodes, and distant metastases accounted for 59.9% (3,049), 82.2% (4,230), and 3.9% (199), respectively. None of the patients underwent surgery and 88.3% (4,491) patients received radiotherapy as the primary treatment. By the end of the follow‐up period, 1646 patients (32.4%) died of TSCC, while 476 (9.4%) died of other causes.

After 15 patients were excluded because of loss to follow‐up or death less than 30 days post treatment, the validation cohort comprised 191 eligible patients. However, all these patients were Chinese and had undergone surgery (Table 1). Their median follow‐up period was 55 (range, 1–119) months, while their median age was 56.0 (range, 18–89) years.

### Nomograms

**Figure 2:**
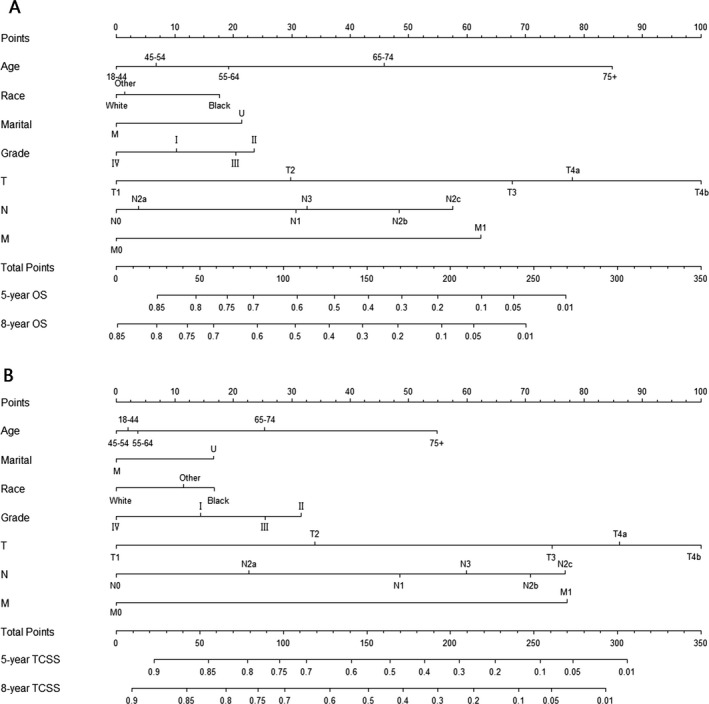
Nomogram for predicting 5‐ and 8‐year (A) overall survival (OS) and (B) tongue cancer‐specific survival (TCSS) in tongue squamous cell carcinoma (TSCC) patients with surgical treatment. The nomogram is used by summing up the points identified on the points scale for each characteristic of the patient. This total point score is then identified at the bottom scale to determine the probability of 5‐ and 8‐year OS or TCSS for an individual patient. Grade: I, Well differentiated; II, Moderately differentiated; III, Poorly differentiated; IV, undifferentiated. Marital: M, married; U, unmarried. Race: Other, American Indian/AK Native, Asian/Pacific Islander.

In the surgery cohort, seven variables were chosen for analysis in the final model based on clinical importance as well as statistical significance for OS probability after univariate and multivariate analyses (Table 2). These variables were age, race, marital status, TNM status, and tumor grade (Fig. S1). A weighted total score calculated from the above variables was used to estimate 5‐ and 8‐year OS for patients with surgical treatment (Fig. 2A). Estimates of cumulative incidences of death from TSCC in the surgery cohort by clinicopathological variables are presented in Table 4. The main factors significantly related to TCSS were age, race, marital status, TNM status, and tumor grade (*P *<* *0.001). A second nomogram predicting TCSS was created using these variables (Fig. 2B).

**Table 2:** Univariate and multivariate analyses of OS in the surgery cohort

Variables	Univariate analysis	Multivariate analysis	
	*P* value	HR (95% CI)	*P* value
Age^a^ (years)	<0.001		
18–44		Reference	
45–54		1.097 (0.936–1.286)	0.251
55–64		1.299 (1.119–1.508)	0.001
65–74		1.865 (1.598–2.177)	<0.001
75+		3.171 (2.710–3.709)	<0.001
Race	<0.001		
White		0.787 (0.674–0.918)	0.002
Black		Reference	
Other^b^		0.801 (0.653–0.918)	0.034
Sex	0.251		
Female			
Male			
Marital status	<0.001		<0.001
Unmarried		1.339 (1.227–1.461)	
Married		Reference	
Grade	<0.001		
I		0.835 (0.742–0.938)	0.002
II		Reference	
III		0.961 (0.873–1.057)	0.441
IV		0.727 (0.401–1.321)	0.296
T stage	<0.001		
T1		Reference	
T2		1.502 (1.361–1.658)	<0.001
T3		2.508 (2.201–2.858)	<0.001
T4a		2.881 (2.510–3.308)	<0.001
T4b		3.861 (2.532–5.887)	<0.001
N stage	<0.001		
N0		Reference	
N1		1.517 (1.355–1.700)	<0.001
N2a		1.051 (0.817–1.353)	0.698
N2b		1.926 (1.717–2.161)	<0.001
N2c		2.180 (1.840–2.583)	<0.001
N3		1.556 (1.110–3.126)	0.010
M stage	<0.001		
M0		Reference	
M1		2.323 (1.726–3.126)	<0.001
Radiation	<0.001		0.931
Yes			
None			

CI; confidence interval; HR, hazard ratio.

a Age at diagnosis.

b Other including American Indian/AK Native, Asian/Pacific Islander.

**Figure 3:**
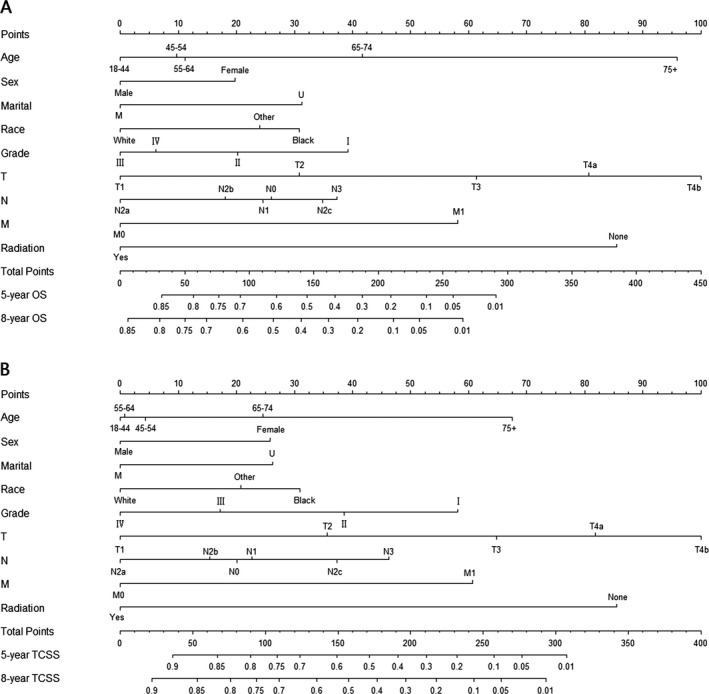
Nomogram for predicting 5‐ and 8‐year (A) overall survival (OS) and (B) tongue cancer‐specific survival (TCSS) in tongue squamous cell carcinoma (TSCC) patients with nonsurgical treatment. The nomogram is used by summing up the points identified on the points scale for each characteristic of the patient. This total point score is then identified at the bottom scale to determine the probability of 5‐ and 8‐year OS or TCSS for an individual patient. Grade: I, Well differentiated; II, Moderately differentiated; III, Poorly differentiated; IV, undifferentiated. Marital: M, married; U, unmarried. Race: Other, American Indian/AK Native, Asian/Pacific Islander.

Briefly, age, race, sex, tumor grade, marital status, TNM status, and radiotherapy were independent risk factors of OS (Table 3 and Fig. S2) and TCSS (Table 4) in the nonsurgery cohort. These independent risk factors were used to develop nomograms for predicting 5‐ and 8‐year OS and TCSS for patients with nonsurgical treatment (Fig. 3A and B).

**Table 3:** Univariate and multivariate analyses of OS in the nonsurgery cohort

Variables	Univariate analysis	Multivariate analysis	
	*P* value	HR (95% CI)	*P* value
Age^a^ (years)	<0.001		
18–44		Reference	
45–54		1.130 (0.891–1.433)	0.313
55–64		1.151 (0.914–1.449)	0.232
65–74		1.702 (1.346–2.152)	<0.001
75+		3.381 (2.659–4.299)	<0.001
Race	<0.001		
White		0.676 (0.594–0.770)	<0.001
Black		Reference	
Other^b^		0.920 (0.729–1.161)	0.481
Sex	<0.001		
Female		Reference	
Male		0.781 (0.702–0.868)	<0.001
Marital status	<0.001		
Unmarried		1.493 (1.364–1.635)	<0.001
Married		Reference	
Grade	<0.001		
I		1.273 (1.094–1.482)	0.002
II		Reference	
III		0.772 (0.704–0.847)	<0.001
IV		0.836 (0.565–1.235)	0.367
T stage	<0.001		
T1		Reference	
T2		1.482 (1.279–1.717)	<0.001
T3		2.190 (1.865–2.572)	<0.001
T4a		2.798 (2.397–3.265)	<0.001
T4b		3.557 (2.793–4.531)	
N stage	<0.001		
N0		Reference	
N1		0.979 (0.859–1.115)	0.750
N2a		0.715 (0.579–0.883)	0.002
N2b		0.902 (0.790–1.030)	0.127
N2c		1.116 (0.980–1.272)	0.098
N3		1.154 (0.943–1.411)	0.165
M stage	<0.001		
M0		Reference	
M1		2.095 (1.761–2.494)	<0.001
Radiation	<0.001		
M0		Reference	
M1		0.341 (0.305–0.381)	<0.001
Radiation	<0.001		
Yes		0.341 (0.305–0.381)	<0.001
None		Reference	

CI; confidence interval; HR, hazard ratio.

a Age at diagnosis.

b Other including American Indian/AK Native, Asian/Pacific Islander.

**Table 4:** Five‐ and eight‐year cumulative incidences of death resulting from TSCC in the surgery cohort and nonsurgery cohort

Variables	CID in surgery cohort			CID in nonsurgery cohort		
	5‐year	8‐year	*P*	5‐year	8‐year	*P*
All patients	0.214	0.223		0.312	0.323	
Age^a^ (years)			<0.001			<0.001
18–44	0.207	0.213		0.330	0.344	
45–54	0.196	0.204		0.298	0.313	
55–64	0.203	0.212		0.261	0.268	
65–74	0.225	0.236		0.315	0.328	
75+	0.255	0.268		0.483	0.491	
Race			<0.001			<0.001
White	0.206	0.216		0.289	0.301	
Black	0.317	0.325		0.490	0.499	
Other^b^	0.226	0.231		0.400	0.400	
Sex			0.566			<0.001
Female	0.217	0.226		0.440	0.445	
Male	0.212	0.222		0.285	0.297	
Marital status			<0.001			<0.001
Unmarried	0.246	0.255		0.387	0.394	
Married	0.200	0.210		0.273	0.286	
Grade			<0.001			<0.001
I	0.133	0.143		0.472	0.487	
II	0.226	0.235		0.358	0.369	
III	0.258	0.269		0.247	0.257	
IV	0.190	0.191		0.222	0.238	
T stage			<0.001			<0.001
T1	0.128	0.138		0.162	0.175	
T2	0.242	0.252		0.252	0.262	
T3	0.388	0.396		0.393	0.403	
T4a	0.456	0.463		0.467	0.476	
T4b	0.566	0.566		0.559	0.559	
N stage			<0.001			<0.001
N0	0.136	0.147		0.340	0.355	
N1	0.292	0.297		0.309	0.319	
N2a	0.191	0.202		0.200	0.208	
N2b	0.366	0.372		0.252	0.261	
N2c	0.447	0.465		0.371	0.382	
N3	0.330	0.341		0.417	0.421	
M stage			<0.001			<0.001
M0	0.210	0.220		0.297	0.308	
M1	0.667	0.683		0.673	0.673	
Radiation			0.012			<0.001
Yes	0.290	0.300		0.273	0.284	
None	0.245	0.254		0.606	0.611	

CID, cumulative incidences of death; TSCC, tongue squamous cell carcinoma.

a Age at diagnosis.

b Other including American Indian/AK Native, Asian/Pacific Islander.

**Figure 4:**
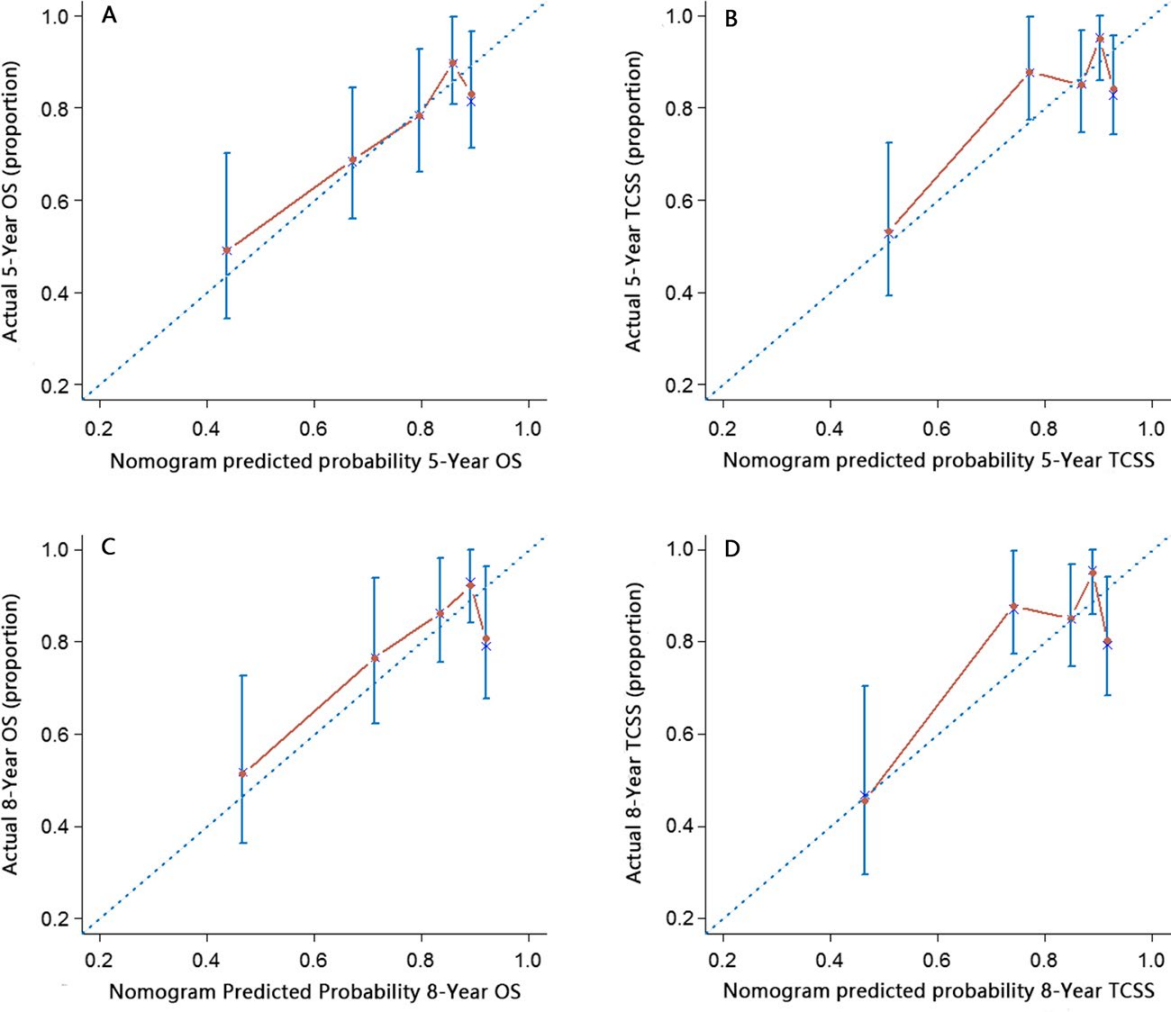
External calibration of the surgery nomogram. (A) 5‐year and (C) 8‐year overall survival nomogram calibration curves; (B) 5‐year and (D) 8‐year tongue cancer‐specific survival nomogram calibration curves. The *X*–axis represents the nomogram–predicted survival, and the actual survival is plotted on the *Y*–axis. The dotted line represents the ideal match between predicted and actual survival. Vertical bars indicate 95% confidence intervals.

### Validation of the nomograms

Validation of the surgery nomogram was conducted both internally and externally. As shown in Table S1, the c‐indexes of the OS and TCSS nomograms in the internal validation cohort (surgery cohort) were 0.709 (95% confidence interval [CI], 0.700–0.719) and 0.728 (95% CI, 0.717–0.739), respectively. In the external validation cohort, the c‐indexes were 0.691(95% CI, 0.677–0.738) and 0.711(95% CI, 0.662–0.760), respectively. The internal and external calibration curves are shown in Figures S3 and S4, respectively, indicating that the nomograms are generally well calibrated.

The nonsurgery nomograms showed excellent performance, with c‐indexes of 0.750 (95% CI, 0.739–0.761) and 0.754 (95% CI, 0.742–0.766) for OS and TCSS, respectively (Table S2). The calibration curves are shown in Figure S4, indicating that the nomograms are generally well calibrated in this case as well.

## Discussion

In this study, we developed nomograms for predicting 5‐ and 8‐year OS and TCSS in patients with TSCC. When externally validated, the surgery nomograms had c‐indexes of 0.691 and 0.711 for OS and TCSS, respectively; these values confirmed the high accuracy of our nomograms.

Compared to TNM staging, which evaluates prognosis based on risk groups, these nomograms have the ability to provide a quantifiable prediction of prognosis per individual patient by incorporating easily available parameters that are important prognostic elements. Consider, for example, the case of a 35‐year‐old White woman diagnosed with TSCC with a tumor size of 2.5 cm and a single 1.5‐cm lymph node in the ipsilateral neck, compared a 70‐year‐old Black man diagnosed with TSCC with tumor size is 3.5 cm and a single 2‐cm lymph node in the ipsilateral neck. Based on the current gold‐standard TNM system, both patients would be classified as T2N1M0 (stage III) with similar outcomes. However, this classification fails to consider the clinicopathological tumor characteristics and patient demographic, resulting in unreliable assessments for individual patients. In contrast, nomograms have the ability to incorporate numerous other factors. The predicted 5‐ and 8‐year OS according to our nomograms were 77%, 51% and 70%, 41%, respectively. These results clearly illustrate the limitations of the TNM classification in predicting individual outcomes. Therefore, the nomogram may be crucial for evaluating patients during the follow‐up period and making treatment decisions, as well as for fine‐tuning and refining the TNM system.

Nomograms are extremely useful tool to facilitate individualized predictions and devise adjuvant treatment plans. To the best of our knowledge, nomograms have previously been constructed to predict the survival of head and neck cancer patients, including those with adenoid cystic carcinoma 18, salivary gland cancer 27, nasopharyngeal cancer 28, and SCC 19, 20, 21. However, this study is the first that aimed at designing a nomogram for TSCC. Shen et al. 21 developed the first nomogram for provide head and neck SCC patients by using the SEER database, which produce a more accurate estimate of their risk of dying from cancer‐specific causes. The TNM stage of TSCC was not registered in the SEER database until 2004; therefore, we searched the database between the years 2004 and 2013 in order to incorporate the actual TNM information from the SEER program data, which is considered one of the larger, high‐quality population‐based cancer registries.

In particular, we also assessed the TCSS using a competing risks model. In this study, the cause of mortality was a growing competing risk because it could alter the probability of death attributed to TSCC 29. Of 4,531 patients in our study who died of various causes, 3,349 (73.9%) died of TSCC, while 1,182 (26.1%) died secondary to other causes; this indicated that a quarter of patients died of causes other than TSCC. Hence, a competing risk nomogram for TSCC is highly useful. Furthermore, to reduce potential bias, we validated the nomograms through calibration plots and performance discrimination using internal and external datasets. As a consequence, our nomograms had extremely powerful predictive abilities, with c‐indexes slightly higher than those of nomograms previously described 18, 19, 20, 21, 27, 28.

Although our nomograms have good accuracy, they also have some limitations. First, they were constructed using retrospective data, which may introduce the risk of treatment selection bias. Therefore, these nomograms must be further validated in a prospective cohort or clinical trial before applying them in clinical practice. Second, information regarding chemotherapy and perineural invasion, which are important prognostic factors for TSCC 30, are not available in the SEER database. Future well‐designed investigations could improve our model by incorporating these variables based on their predictive power. Third, different surgery modalities (i.e., resection of the primary tumor only or with the inclusion of lymphadenectomy) were also an important factor and may be related to patients’ outcomes. However, the SEER database only records the “reason no cancer‐directed surgery”, that is, the reason that surgery was not performed on the primary site. Therefore, we divided the SEER‐extracted patients into the surgery and nonsurgery cohorts, and established respective prognostic models for each. Lastly, although we performed external validation for surgery nomograms with a good performance, our validation cohort consisted of patients from a single hospital database in Asia. We are therefore planning additional validation on studies using multi‐institutional international datasets.

In conclusion, we used a large population‐based cohort to create a series of nomograms that estimated 5‐ and 8‐year OS and TCSS for patients with TSCC for the first time. These nomograms demonstrated an accurate and powerful predictive ability as shown by internal and external validation. These accurate and easy‐to‐use tools can be used to facilitate the counseling of patients regarding their prognoses, and to contribute to treatment decision‐making for the management of patients with TSCC.

## Conflict Of Interest

The authors declare no conflicts of interest.

## Supporting information


**Figure S1.** Overall Kaplan–Meier survival estimates by age, marital status, race, TNM and grade groups in surgery cohort, respectively.

**Figure S2.** Overall Kaplan–Meier survival estimates by age, race, sex, TNM, marital status, grade and radiation groups in non‐surgery cohort, respectively.

**Figure S3. Internal calibration of the surgery nomogram**. (A) 5‐year and (C) 8‐year overall survival (OS) nomogram calibration curves; (B) 5‐year and (D) 8‐year tongue cancer‐specific survival (TCSS) nomogram calibration curves. The X–aixs represents the nomogram–predicted survival, and the actual survival is plotted on the Y–axis. The dotted line represents the ideal match between predicted and actual survival. Vertical bars indicate 95% confidence intervals.

**Figure S4. Internal calibration of the non‐surgery nomogram**. (A) 5‐year and (C) 8‐year overall survival (OS) nomogram calibration curves; (B) 5‐year and (D) 8‐year tongue cancer‐specific survival (TCSS) nomogram calibration curves. The X–aixs represents the nomogram–predicted survival, and the actual survival is plotted on the Y–axis. The dotted line represents the ideal match between predicted and actual survival. Vertical bars indicate 95% confidence intervals.

**Table S1**: The c‐index for the nomogram to predict overall survival and tongue cancer‐specific survival in surgery cohort.
