# Supplementary figures and images for: Nomograms to estimate long‐term overall survival and tongue cancer‐specific survival of patients with tongue squamous cell carcinoma
Source: Cancer Med. 2017 Apr 14;6(5):1002–13. doi: 10.1002/cam4.1021 (PMC5430099; doi:10.1002/cam4.1021)

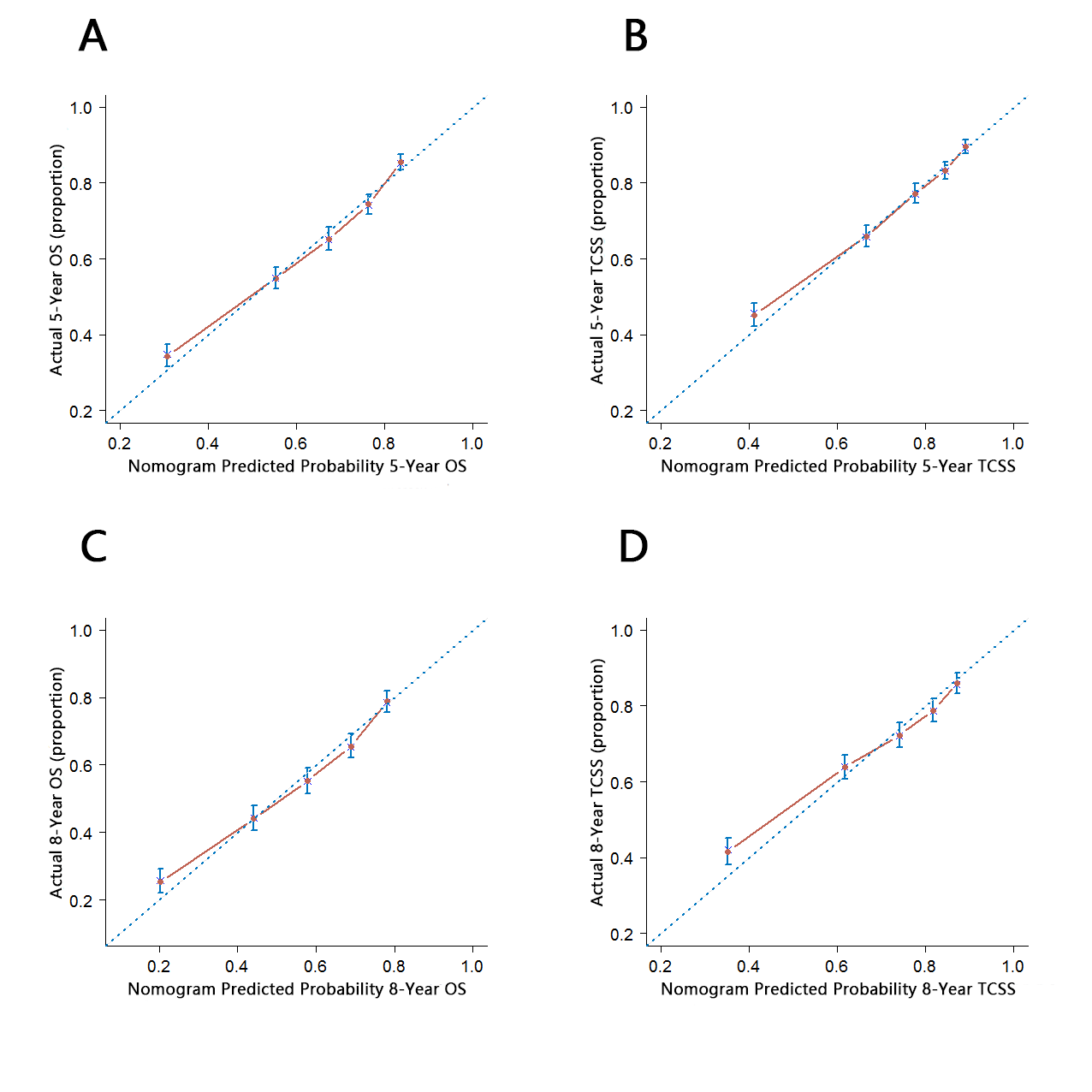

Supplement: Supplementary file 1 — Figure S1. Overall Kaplan–Meier survival estimates by age, marital status, race, TNM and grade groups in surgery cohort, respectively. [file CAM4-6-1002-s001.tif]

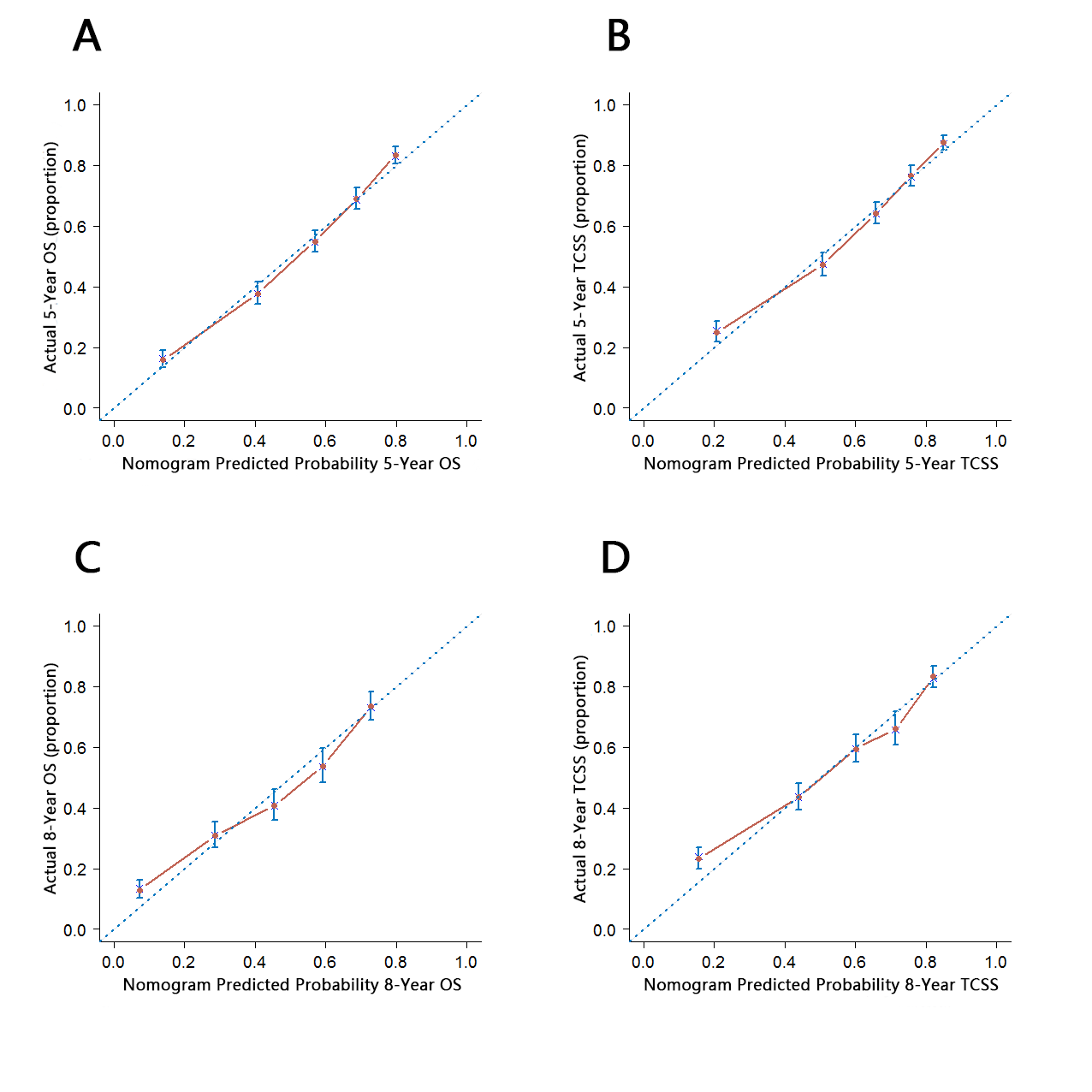

Supplement: Supplementary file 2 — Figure S2. Overall Kaplan–Meier survival estimates by age, race, sex, TNM, marital status, grade and radiation groups in non‐surgery cohort, respectively. [file CAM4-6-1002-s002.tif]

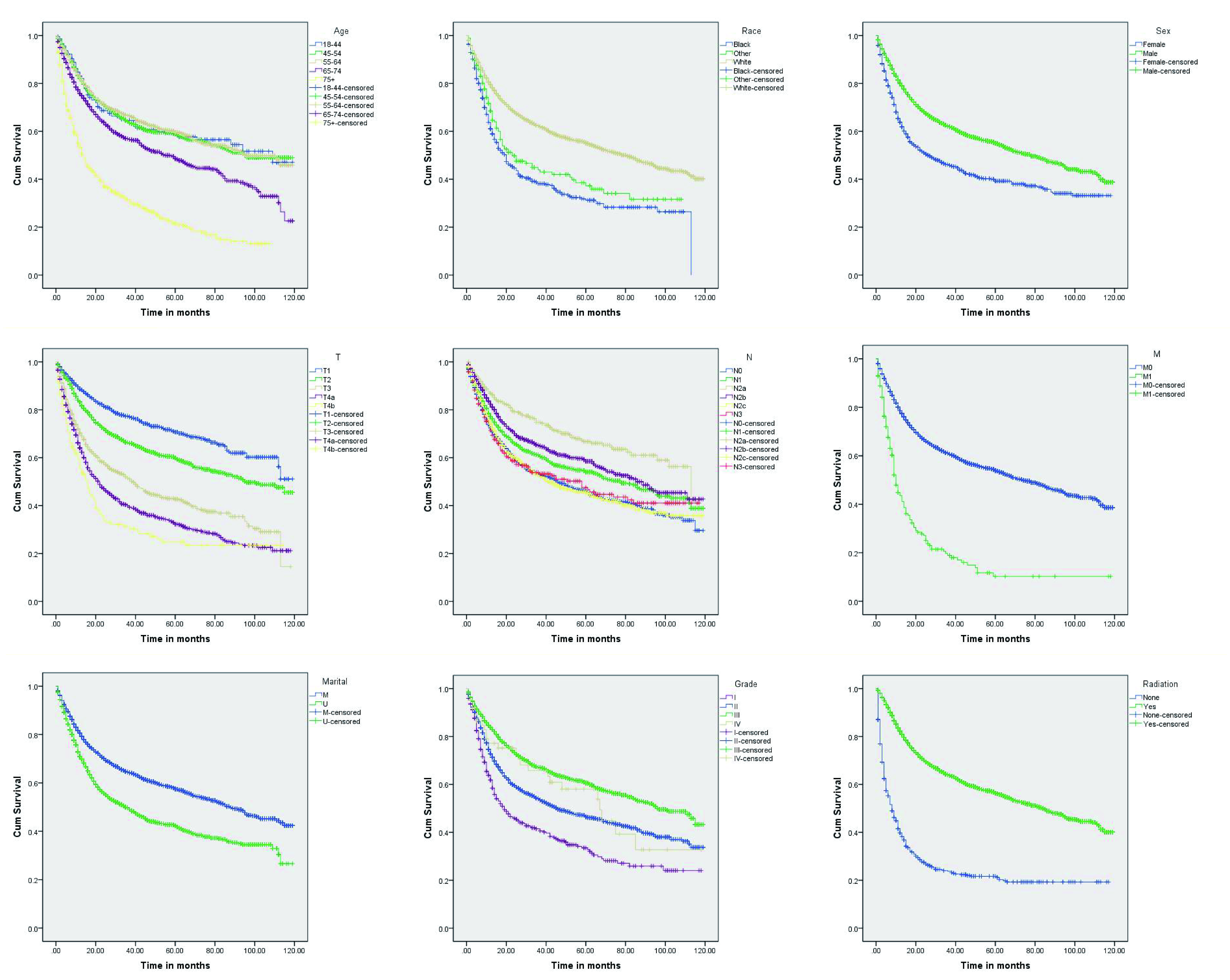

Supplement: Supplementary file 3 — Figure S3. Internal calibration of the surgery nomogram. (A) 5‐year and (C) 8‐year overall survival (OS) nomogram calibration curves; (B) 5‐year and (D) 8‐year tongue cancer‐specific survival (TCSS) nomogram calibration curves. The X–aixs represents the nomogram–predicted survival, and the actual survival is plotted on the Y–axis. The dotted line represents the ideal match between predicted and actual survival. Vertical bars indicate 95% confidence intervals. [file CAM4-6-1002-s003.jpg]

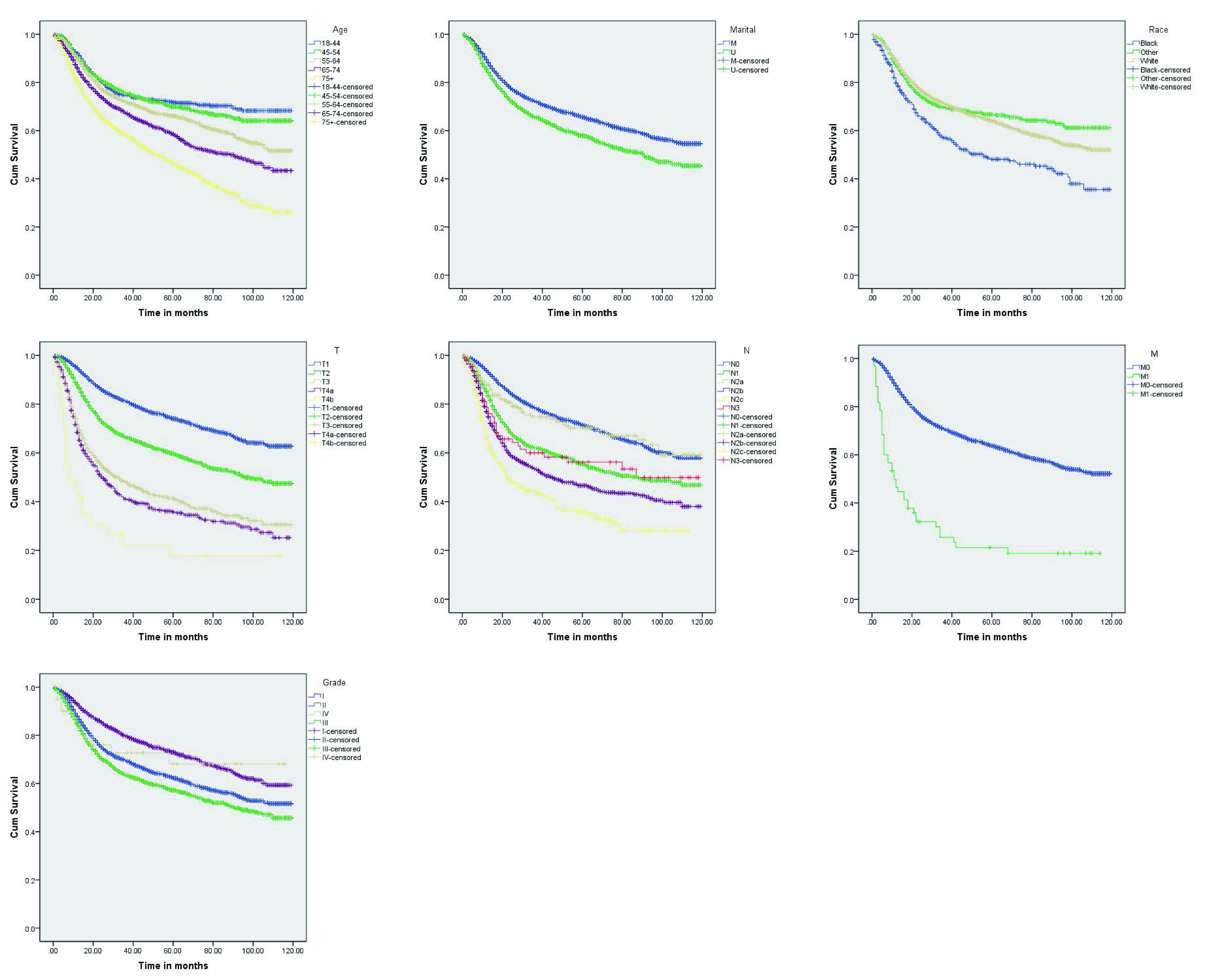

Supplement: Supplementary file 4 — Figure S4. Internal calibration of the non‐surgery nomogram. (A) 5‐year and (C) 8‐year overall survival (OS) nomogram calibration curves; (B) 5‐year and (D) 8‐year tongue cancer‐specific survival (TCSS) nomogram calibration curves. The X–aixs represents the nomogram–predicted survival, and the actual survival is plotted on the Y–axis. The dotted line represents the ideal match between predicted and actual survival. Vertical bars indicate 95% confidence intervals. [file CAM4-6-1002-s004.jpg]
