# Supplementary material for: Nomograms to estimate long‐term overall survival and tongue cancer‐specific survival of patients with tongue squamous cell carcinoma
Source: Cancer Med. 2017 Apr 14;6(5):1002–13. doi: 10.1002/cam4.1021 (PMC5430099; doi:10.1002/cam4.1021)
Supplement: Supplementary file 5 — Table S1: The c‐index for the nomogram to predict overall survival and tongue cancer‐specific survival in surgery cohort. [file CAM4-6-1002-s005.docx]

**Supplemental** **Table 1**: The c-index for the nomogram to predict OS and TCSS in Surgery Cohort

| \| **Groups** \| \| --- \| | **OS**  HR 95%CI | | **TCSS**  HR 95%CI | |
| --- | --- | --- | --- | --- | --- |
| Surgery Cohort | 0.709 | 0.700-0.719 | 0.728 | 0.717-0.739  0.662-0.760 |
| Validation Cohort | 0.691 | 0.644-0.738 | 0.711 |  |

Abbreviations: OS, overall survival; TCSS, tongue squamous cell carcinoma cancer-specific survival; HR, hazard ratio; CI, confidence interval.
